# The intersectional effect of poverty, home ownership, and racial/ethnic composition on mean childhood blood lead levels in Milwaukee County neighborhoods

**DOI:** 10.1371/journal.pone.0234995

**Published:** 2020-06-19

**Authors:** Emily E. Lynch, Helen C. S. Meier

**Affiliations:** Joseph J. Zilber School of Public Health University of Wisconsin- Milwaukee, Milwaukee, Wisconsin, United States of America; University of Botswana, BOTSWANA

## Abstract

Environmental conditions that contribute to childhood lead exposure are spatially patterned. Socioeconomic and racial inequities in childhood lead exposure have been well documented, however childhood lead exposure in Milwaukee is understudied. As a segregated rustbelt metropolitan area with childhood lead exposure concerns, Milwaukee is uniquely positioned to evaluate the synergistic effects of racial and economic drivers of childhood lead exposure. Using surveillance data from the Wisconsin Department of Health Services, Division of Public Health and the US Census Bureau, this cross-sectional study determined the intersectional effect of poverty, home ownership, and racial/ethnic composition on childhood lead exposure in Milwaukee County neighborhoods using linear regression adjusting for average census tract housing age and number of children. The final analytical sample consisted of 48,393 individual childhood blood lead levels aggregated to 215 Milwaukee County census tracts. Census tracts with mean childhood blood lead levels greater than or equal to 5 μg/dL were predominantly low home ownership, high poverty, and majority non-White census tracts. The effects of low home ownership, high poverty, and majority non-White census tracts were synergistic, producing 1.78 (95% CI: 1.44, 2.11) μg/dL higher mean childhood blood lead level than high home ownership, low poverty, and majority White census tracts (referent). This research reveals that social determinants at the neighborhood level co-occur and interact to produce inequities in childhood lead exposure. Lead prevention efforts should align with equity-focused housing and economic policies that target primary prevention in neighborhoods disproportionately burdened by childhood lead exposure.

## Introduction

Childhood lead poisoning continues to be a significant public health concern throughout the United States despite federal regulations to eliminate lead from paint, gasoline, and other consumer products [1]. Lead is a persistent and pervasive contaminant that is found in paint, dust, soil, potable water piping, and air [1]. While the Centers for Disease Control and Prevention (CDC) classifies childhood blood lead levels at or above 5 μg/dL as “elevated”, there is no safe level of lead exposure [2]. Even low levels of lead exposure in children can interfere with the brain’s ability to develop, resulting in neurological and developmental delays [1,3,4]. Childhood lead exposure can cause irreversible brain damage and is associated with various adverse physical, mental, and behavioral outcomes [3,4].

Environmental conditions that contribute to childhood lead exposure are spatially patterned. Childhood lead exposure disproportionately impacts neighborhoods with low socioeconomic characteristics [5–9], older housing stock [7,10], neighborhoods of color [5–7,11], and neighborhoods in geographic proximity to industrial lead emissions [12,13]. Older metropolitan areas in the US, such as Milwaukee County, Wisconsin, share a disproportionate burden of lead exposure due to housing stock age and the legacy of infrastructure material choices made a hundred years ago. According to 5-year American Community Survey Estimates between 2012 and 2016, 84% of occupied housing in Milwaukee County was built in 1979 or before and 40% was built before 1950 [14]. Additionally, more than 75,000 homes are serviced by lead water laterals [15]. However, the presence of lead in homes and infrastructure alone do not determine whether children are exposed to lead; childhood lead exposure is influenced by neighborhood social context driven by power differentials and institutional racism. Neighborhood racial and economic inequality is actively maintained through racist housing and economic policies and practices at local, state, and federal levels. Milwaukee is commonly ranked among the most segregated major metropolitan areas in the United States [16], has areas of intense socioeconomic deprivation [17], and faces challenges combating childhood lead exposure. Indeed, according to the Wisconsin Department of Health Services in 2016, among children under the age of six that were tested for blood lead levels, 8.8% of children in Milwaukee County, the most populous county in the state, had blood lead levels greater than or equal to 5 μg/dL, as compared to 5.0% of children in Wisconsin [18].

Identifying social determinants of childhood lead exposure that co-occur guides primary prevention efforts by understanding holistically the characteristics of neighborhoods most at-risk. Exploring the contribution of single sociodemographic neighborhood risk factors on health inequities is methodologically complex due to the spatial patterning of racial and economic segregation and the numerous pathways segregation operates through to influence health [19–21]. Further, this study examines “upstream” social drivers of childhood lead exposure because case management and environmental assessments to mitigate lead exposure for children residing in the City of Milwaukee are rationed to children with one venous blood lead test ≥ 20 μg/dL or two venous blood lead tests ≥ 15 μg/dL drawn at least 90 days apart [22].

Socioeconomic and racial inequities in childhood lead exposure have been well documented [5–11, 23–27], however, drivers of childhood lead exposure in Milwaukee is understudied. Milwaukee is uniquely positioned to evaluate synergistic effects of neighborhood sociodemographic characteristics on childhood lead exposure due to the socioeconomic and racial patterning exacerbated by the lack of formal lead prevention infrastructure. As a segregated rustbelt metropolitan area with childhood lead exposure concerns, Milwaukee is, unfortunately, well-suited to examine the intersection of home ownership, poverty, and racial/ethnic composition on neighborhood childhood lead exposure risk. Using surveillance data from the Wisconsin Department of Health Services, Division of Public Health and the US Census Bureau, this cross-sectional study tested the hypothesis that the combined effect of low home ownership, high poverty, and majority non-White racial/ethnic composition would result in the highest risk for childhood blood lead levels in Milwaukee County neighborhoods.

## Materials and methods

### Data

Individual blood lead level surveillance data for children six years old or younger residing in Milwaukee County, Wisconsin were obtained from the Wisconsin Department of Health Services, Division of Public Health for 2014–2016 (N = 57,268). Individual blood lead levels were aggregated to generate Milwaukee County census tract mean childhood blood lead levels (N = 296). Census tract mean childhood blood lead level data was merged with socioeconomic and demographic data obtained from the US Census Bureau using census tracts. Census tracts with no socioeconomic or demographic data available were subsequently dropped from the analysis, resulting in 48,393 individual blood lead levels aggregated to the final census tract-level analytical sample of 215. The average number of individual childhood blood lead observations contributing to census tract-level means was 225; the minimum number of individual childhood blood lead observations contributing to a census tract-level mean was six. This study was approved by the University of Wisconsin—Milwaukee Institutional Review Board.

#### Blood lead level data

Blood lead level surveillance data were obtained from routine healthcare appointments. Data included individuals’ sex, age, race/ethnicity, maximum blood lead level from their highest value blood test between 2014 and 2016, the type of blood sample taken (capillary, venous, or unknown), the month and year the test was administered, and the census tract in which the individual resided at the time of the blood test. To account for variation in detection levels of testing methods, all individual blood lead levels of 1.0 μg/dL were divided by the square root of 2 before aggregating to the census tract level [5,6,28]. For descriptive statistics, census tract mean childhood blood lead levels were categorized as less than 5 μg/dL or ≥ 5 μg/dL, consistent with the Centers for Disease Control and Preventions reference level for childhood lead exposure [2]. All remaining analyses utilized continuous mean census tract childhood blood lead levels.

#### Neighborhood socioeconomic and demographic characteristics

Data on home ownership, poverty, and racial/ethnic composition were obtained from the US Census Bureau, American Community Survey (ACS) 2012–2016 5-year estimates for each Milwaukee County census tract [29–31]. This study emulates previous research on childhood lead exposure using census tracts as a proxy for neighborhoods [5,6,8]. The optimum size of a single census tract is approximately 4,000 people, representing small, relatively permanent statistical subdivisions that provide stable geographic units for presenting statistical data [32]. Additionally, census tracts have been utilized to monitor area based socioeconomic characteristics and health to illustrate the context of social patterning [33,34].

Home ownership was measured using the percent of owner-occupied housing units in each census tract. Poverty was measured using the percent of families in the census tract whose income in the last 12 months was below the United States poverty level threshold, determined by the US Census Bureau, incorporating the number of people in family, age of its members, and number of children under 18 years related to householder. Home ownership and poverty levels were dichotomized into low and high according to the mean value among census tracts (i.e., a mean split of the census-tract data). High ownership was categorized as census tracts with ≥ 40% of occupied housing owned and low ownership was categorized as census tracts with < 40% of occupied housing owned. High poverty was categorized as census tracts with ≥ 25% of families living below the poverty level and low poverty was categorized as census tracts with < 25% of families living below the poverty level. Dichotomizing the poverty level data at the mean split value of 25% falls directly between the US Census Bureau’s categorization of concentrated poverty as census tracts with a poverty rate of 20% or more [35], and the commonly utilized cut-off of 30% for areas of extreme concentrated poverty [36,37]. Majority White and majority non-White census tracts were categorized using census tract racial demographic estimates. Census tracts with ≥ 50% of residents who identified as non-Hispanic White were classified as majority White and census tracts with < 50% of residents that identified as non-Hispanic White were classified as majority non-White. The categorization of majority Non-Hispanic White vs. Majority non-White neighborhoods is supported by previous literature [38,39] and the bifurcation model which posits that neighborhoods adhere to a White/non-White color line as a means to reinforce racial hierarchy [40].

### Covariates

Several variables were used to characterize census tracts in this analysis, including census tract educational attainment, housing age, and number of children. Data on educational attainment and housing age were obtained from the US Census Bureau, ACS 2012–2016 5-year estimates for each Milwaukee County census tract [14,41]. Neighborhood educational attainment was dichotomized into census tracts with ≥ 50% of the population 25 years and older with a high school diploma/equivalent or less education (includes no schooling, nursery school, kindergarten, and grade 1–12 but no high school diploma) or census tracts with < 50% of the population 25 years and older with a high school diploma/equivalent or less education. Housing age was dichotomized into census tracts with ≥ 50% of the housing built prior to 1950 or census tracts with < 50% of the housing built prior to 1950.

Data from the US Census Bureau, 2010 Decennial Census were obtained to estimate the number of children age six years and under that resided in Milwaukee County census tracts [42]. The estimates for the number of children per census tract was treated as a continuous variable for analysis. Childhood lead testing penetrance estimates were calculated using the number of children six years of age and under tested in each Milwaukee County census tract between 2014 and 2016 divided by the number of children six years of age and under residing in each census tract as reported in the 2010 Decennial Census.

### Statistical analysis

Descriptive statistics were conducted to evaluate characteristics of Milwaukee County census tracts by elevated mean childhood blood lead level status (census tracts with mean childhood blood lead levels ≥ 5μg/dL or < 5μg/dL) and by housing tenure and poverty levels. Differences in observed distributions of characteristics were evaluated using two-tailed T-tests, Wilcoxon two-sample tests, and F-tests for average tract continuous variables and chi-square tests for categorical variables. We further limited the analysis to census tracts with high poverty, low home ownership (high socioeconomic disadvantage) and low poverty, high home ownership (low socioeconomic disadvantage) (n = 168) for regression analysis. The intersectional effect of home ownership, poverty, and race/ethnicity on census tract mean childhood blood lead levels was estimated using linear regression. Covariates were selected for model inclusion based on *a priori* knowledge of potential confounding variables and the covariate’s association with the exposure and outcome; the final covariates were census tract-level housing age and number of children. A two-sided p-value <0.05 was considered statistically significant. All analyses were conducted in SAS 9.4 (SAS Institute, Inc., Cary, NC).

## Results

**Fig 1:**
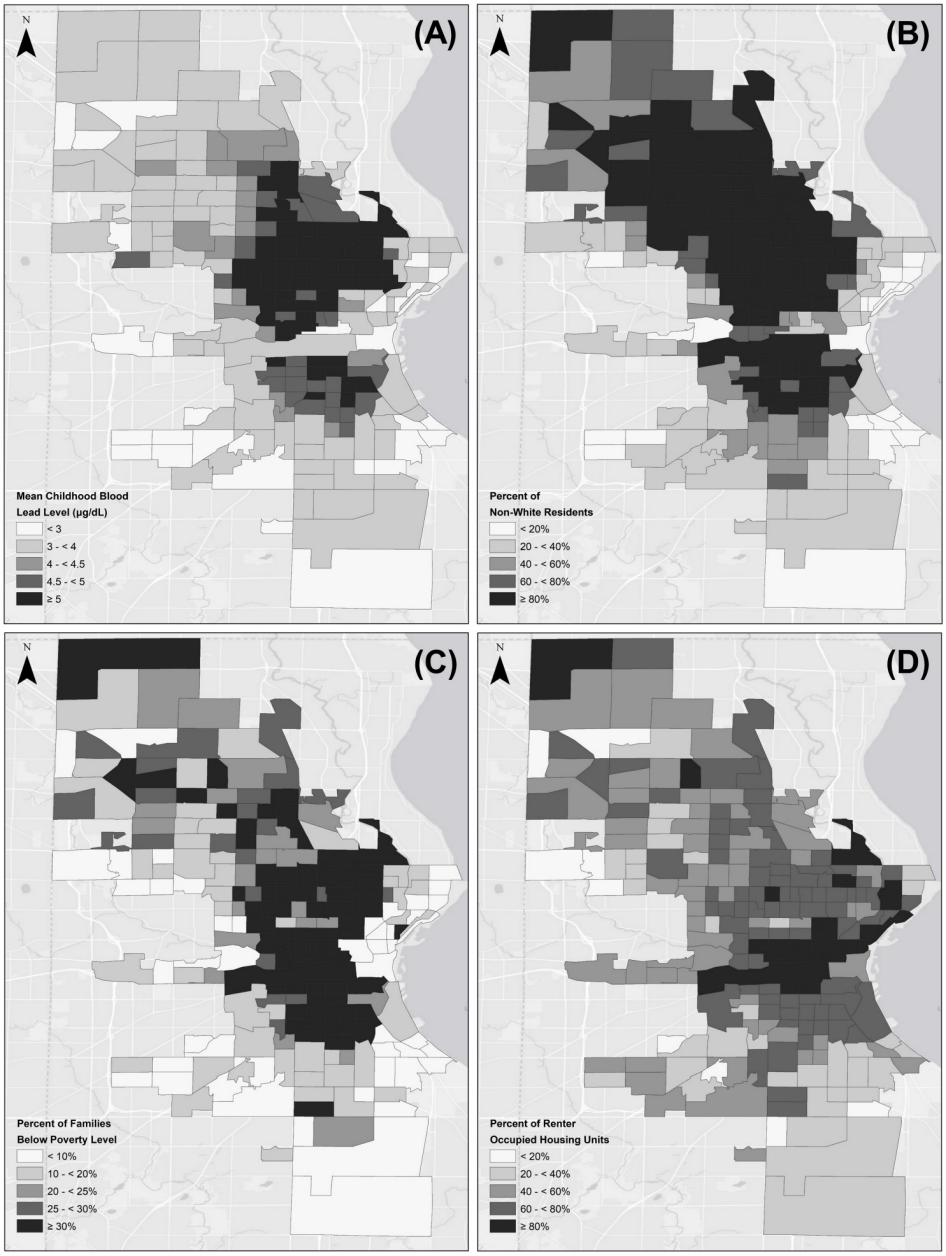
Mean childhood blood lead levels, socioeconomic, and racial/ethnic composition of Milwaukee County census tracts (n = 215). Milwaukee County census tract mean (A) childhood blood lead levels (B) percent non-White residents (C) percent of families living below the poverty level (D) percent of renter occupied housing units. Manual breaks (5 classes) were utilized to display meaningful breaks in the data based on literature and relevant levels for policy and health action. Data was obtained from the 2014–2016 Lead Surveillance Data from the Wisconsin Department of Health Services, Division of Public Health and the U.S. Census Bureau, 2012–2016 American Community Survey 5-Year Estimates. Service layer credits to Esri, HERE, Garmin, (c) OpenStreetMap contributors, and the GIS user community.

Utilizing 2010 Decennial Census child population estimates, the mean childhood lead testing penetrance for the 215 Milwaukee County census tracts analyzed was estimated to be 68.68%. The mean childhood lead testing penetrance for census tracts within the City of Milwaukee was higher (69.32%) than the childhood lead testing penetrance for census tracts outside the City of Milwaukee (46.40%). Among all 215 Milwaukee County census tracts, the average census tract mean childhood blood lead level was 4.33 μg/dL. Fig 1 depicts the spatial distribution of census tract mean childhood blood lead levels, percent non-White residents, percent renter occupied housing, and percent of families living below the poverty level in Milwaukee County. Fig 1 reveals a pattern of geographic disadvantage in which areas with a high concentration of elevated census tract mean childhood blood lead levels, predominantly on the North and South side of Milwaukee, align with areas of high poverty, low home ownership, and areas with predominantly non-White residents.

Descriptive statistics for Milwaukee County census tracts by dichotomized census tract mean childhood blood lead levels are displayed in Table 1. Sixty (28%) of the 215 census tracts in Milwaukee County had a mean childhood blood lead level at or above the 5 μg/dL Center for Disease Control and Prevention level of concern and were classified as “elevated”. These elevated census tracts had, on average, a higher proportion of non-Hispanic Black or African American residents, residents who rent, and residents who live below the federal poverty line compared to census tracts with mean childhood blood lead levels less than 5 μg/dL (S1 Table). Among elevated census tracts, an average of 67% of residents identified as non-Hispanic Black or African American, 22% identified as Hispanic or Latino or other, and 11% identified as non-Hispanic White (S1 Table). Ninety-seven percent of elevated census tracts were categorized as majority non-White (n = 58) while only 3% of elevated census tracts were categorized as majority White (n = 2) (Table 1). Eighty-eight percent of elevated census tracts (n = 53) were categorized as high poverty census tracts, while 69% of not elevated census tracts were categorized as low poverty census tracts (n = 107) (Table 1). Eighty-five percent of elevated census tracts were categorized as low home ownership census tracts (n = 51) and 61% of not elevated census tracts were categorized as high home ownership census tracts (n = 94) (Table 1). In addition, 93% of elevated census tracts had the majority of housing stock built before 1950 (n = 56) and 87% of elevated census tracts were categorized as low educational attainment (n = 52) (Table 1).

**Table 1:** Descriptive statistics of Milwaukee County census tracts by mean childhood blood lead level (n = 215).

	“Elevated” Census tracts with mean childhood blood lead level ≥ 5μg/dL	“Not Elevated” Census tracts with mean childhood blood lead level < 5μg/dL	p-value^a^
	N = 60	N = 155	
	Mean (Standard Deviation) or N (Percent)		
***Wisconsin Surveillance Data***			
Childhood Blood Lead Level (μg/dL)	6.09 (0.81)	3.65 (0.70)	<0.0001
***2012–2016 American Community Survey 5-Year Estimates***			
Neighborhood Racial/Ethnic Composition			<0.0001
Majority White^b^	2 (3.33%)	74 (47.74%)	
Majority non-White^c^	58 (96.67%)	81 (52.26%)	
Neighborhood Poverty Level			<0.0001
High Poverty^d^	53 (88.33%)	48 (30.97%)	
Low Poverty^e^	7 (11.67%)	107 (69.03%)	
Neighborhood Housing Tenure			<0.0001
High Home Ownership^f^	9 (15.00%)	94 (60.65%)	
Low Home Ownership^g^	51 (85.00%)	61 (39.35%)	
Neighborhood Educational Attainment			<0.0001
Low Educational Attainment^h^	52 (86.67%)	51 (32.90%)	
High Educational Attainment^i^	8 (13.33%)	104 (67.10%)	
Neighborhood Housing Age			<0.0001
Majority of Housing Built Before 1950^j^	56 (93.33%)	67 (43.23%)	
Majority of Housing Built 1950 or After^k^	4 (6.67%)	88 (56.77%)	
***US Census Bureau 2010 Decennial Census Data***			
Children 6 Years and Under	13.30%	10.10%	<.0001

^a^p-value from two-tailed t-test, Wilcoxon two-sample test, or chi-square test

^b^Census tracts with ≥ 50% of non-Hispanic White residents

^c^Census tracts with < 50% of non-Hispanic White residents

^d^Census tracts with ≥ 25% of families living below poverty level

^e^Census tracts with < 25% of families living below poverty level

^f^Census tracts with ≥ 40% of occupied housing that is owned

^g^Census tracts with < 40% of occupied housing that is owned

^h^Census tracts with ≥ 50% of the population 25 years and older with a high school diploma/equivalent or less education (including no schooling, nursery school, kindergarten, and grade 1–12 but no high school diploma)

^i^Census tracts with or < 50% of the population 25 years and older with a high school diploma/equivalent or less education (including no schooling, nursery school, kindergarten, and grade 1–12 but no high school diploma)

^j^Census tracts with ≥ 50% of the housing built before 1950

^k^Census tracts with < 50% of the housing built before 1950

Descriptive statistics for Milwaukee County census tracts by intersection of housing tenure and poverty levels are displayed in Table 2. Census tracts with high home ownership and low poverty had the lowest mean childhood blood lead level (3.59 μg/dL), while census tracts with low home ownership and high poverty had the highest mean childhood blood lead level (5.23 μg/dL) (Table 2). Sixty-two percent of high home ownership, low poverty census tracts were majority White (n = 53) and 94% of low home ownership, high poverty census tracts were majority non-White (n = 78) (Table 2).

**Table 2:** Descriptive statistics of Milwaukee County census tracts by intersection of housing tenure and poverty level (n = 215).

	Census tracts with High Home Ownership^a^ & Low Poverty^c^	Census tracts with Low Home Ownership^b^ & Low Poverty^c^	Census tracts with High Home Ownership^a^ & High Poverty^d^	Census tracts with Low Home Ownership^b^ & High Poverty^d^	p-value^e^
	N = 85	N = 29	N = 18	N = 83	
	Mean (Standard Deviation) or N (Percent)				
***Wisconsin Surveillance Data***					
Childhood Blood Lead Level (μg/dL)	3.59 (0.86)	3.78 (1.25)	4.58 (1.07)	5.23 (1.21)	<0.0001
***2012–2016 American Community Survey 5-Year Estimates***					
Neighborhood Racial/Ethnic Composition					<0.0001
Majority White^f^	53 (62.35%)	18 (62.07%)	0 (0.00%)	5 (6.02%)	
Majority Non-White^g^	32 (37.65%)	11 (37.93%)	18 (100.00%)	78 (93.98%)	
Neighborhood Educational Attainment					<0.0001
Low Educational Attainment^h^	13 (15.29%)	11 (37.93%)	14 (77.78%)	65 (78.31%)	
High Educational Attainment^i^	72 (84.71%)	18 (62.07%)	4 (22.22%)	18 (21.69%)	
Neighborhood Housing Age					0.0004
Majority of Housing Built Before 1950^j^	35 (41.18%)	17 (58.62%)	10 (55.56%)	61 (73.49%)	
Majority of Housing Built 1950 or After^k^	50 (58.82%)	12 (41.38%)	8 (44.44%)	22 (26.51%)	
***US Census Bureau 2010 Decennial Census Data***					
Children 6 Years and Under	9.99%	6.51%	12.73%	13.22%	<0.0001

^a^Census tracts with ≥ 40% of occupied housing that is owned

^b^Census tracts with < 40% of occupied housing that is owned

^c^Census tracts with < 25% of families living below poverty level

^d^Census tracts with ≥ 25% of families living below poverty level

^e^ p-value from f-test or chi-square test

^f^Census tracts with ≥ 50% of non-Hispanic White residents

^g^Census tracts with < 50% of non-Hispanic White residents

^h^Census tracts with ≥ 50% of the population 25 years and older with a high school diploma/equivalent or less education (including no schooling, nursery school, kindergarten, and grade 1–12 but no high school diploma)

^i^Census tracts with or < 50% of the population 25 years and older with a high school diploma/equivalent or less education (including no schooling, nursery school, kindergarten, and grade 1–12 but no high school diploma)

^j^Census tracts with ≥ 50% of the housing built before 1950

^k^Census tracts with < 50% of the housing built before 1950

**Fig 2:**
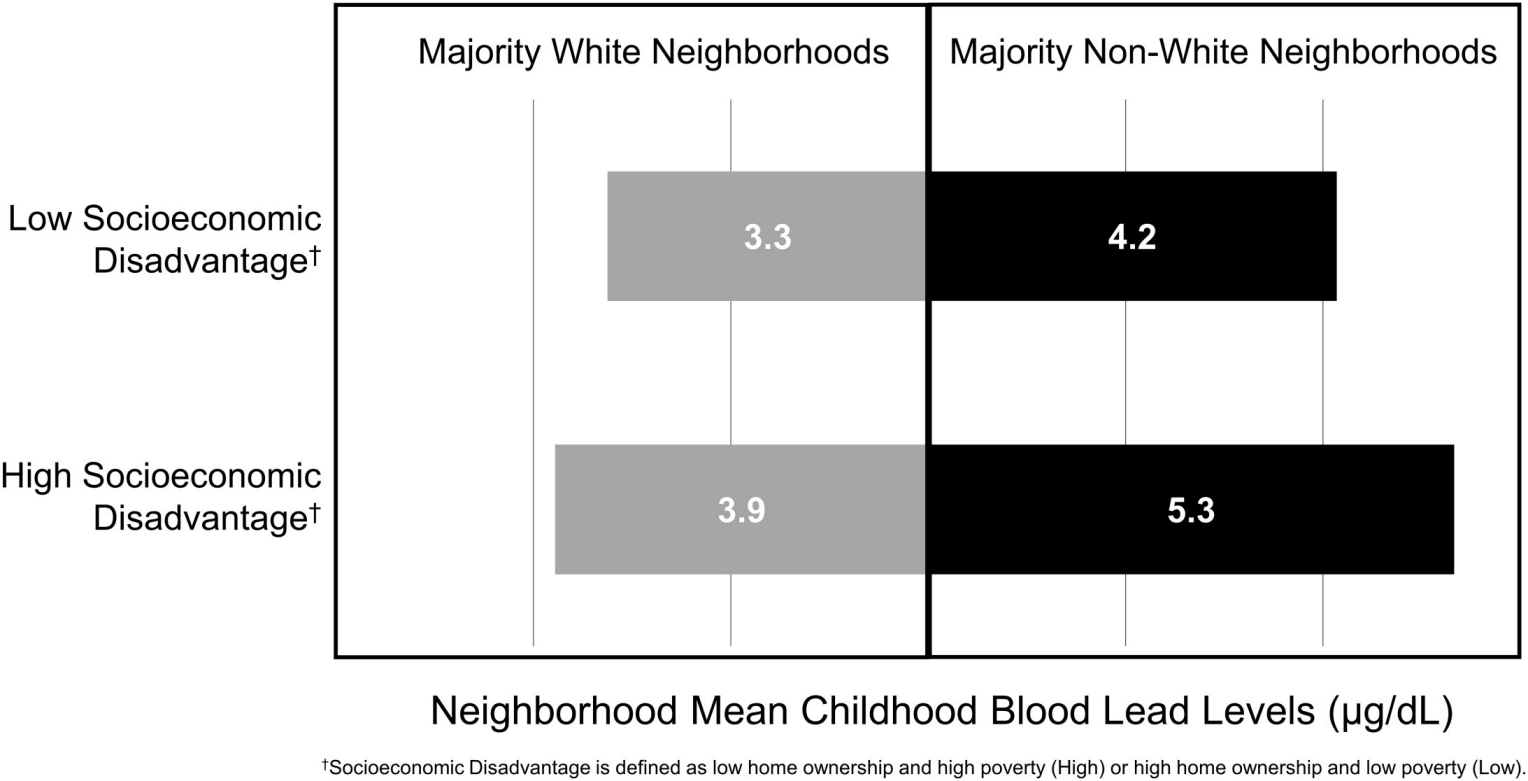
Mean childhood blood lead levels by racial/ethnic composition and socioeconomic disadvantage among Milwaukee County neighborhoods (n = 215). Data was obtained from the 2014–2016 Lead Surveillance Data from the Wisconsin Department of Health Services, Division of Public Health and U.S. Census Bureau, 2012–2016 American Community Survey 5-Year Estimates.

Elevated census tracts were predominantly low home ownership, high poverty and majority non-White (S3 Table). Among the 58 majority non-White census tracts with a mean childhood blood lead level ≥ 5 μg/dL, 81% were low home ownership and high poverty census tracts (S3 Table). Milwaukee County mean census tract childhood blood lead levels were highest among majority non-White census tracts with high socioeconomic disadvantage (5.33 μg/dL) and lowest among majority White census tracts with low socioeconomic disadvantage (3.25 μg/dL) (Fig 2).

Linear regression results are displayed in Table 3. Among high home ownership and low poverty census tracts (isolating the effect of race/ethnicity), majority non-White census tracts had a 0.85 μg/dL (95% Confidence Interval [CI]: 0.46, 1.24; df = 6, p<0.001) higher average childhood blood lead level than majority White tracts after adjusting for census tract housing age and number of children. Further, among majority White census tracts, low home ownership and high poverty census tracts (isolating the effect of socioeconomic disadvantage), had a 0.37 μg/dL (95% CI: -0.42, 1.16; df = 6, p = 0.356) higher mean childhood blood lead level than high home ownership and low poverty tracts after adjusting for average census tract housing age and number of children. When the three exposures were assessed jointly, low home ownership, high poverty, and majority non-White census tracts had a 1.78 (95% CI: 1.44, 2.11; df = 6, p<0.0001) μg/dL higher mean childhood blood lead level than high home ownership, low poverty, and majority White census tracts, after adjusting for average census tract housing age and number of children.

**Table 3:** Adjusted linear regression estimating association of socioeconomic disadvantage and racial ethnic composition on mean childhood blood lead level among Milwaukee County census tracts (n = 168).

	Change in Census Tract Mean Childhood Blood Lead Level (μg/dL) (95% Confidence Intervals)		
	Model 1	Model 2	Model 3
Low Socioeconomic Disadvantage^a^ & Majority White^b^	Ref.	Ref.	Ref.
High Socioeconomic Disadvantage^c^ & Majority White^b^	0.53 (-0.37, 1.42)	0.48 (-0.30, 1.27)	0.37 (-0.42, 1.16)
Low Socioeconomic Disadvantage^a^ & Majority Non-White^d^	0.89* (0.46, 1.32)	0.75* (0.37, 1.12)	0.85* (0.46, 1.24)
High Socioeconomic Disadvantage^c^ & Majority Non-White^d^	2.08* (1.74, 2.42)	1.67* (1.35, 1.99)	1.78* (1.44, 2.11)

Model 1 unadjusted

Model 2 adjusted for average census tract housing age

Model 3 additionally adjusted for average number of children 6 years of age and under per census tract

*p-value < 0.05

^a^Census tracts with high home ownership (≥ 40% of occupied housing that is owned) and low poverty (< 25% of families living below poverty level)

^b^Census tracts with ≥ 50% of non-Hispanic White residents

^c^Census tracts with low home ownership (< 40% of occupied housing that is owned) and high poverty (≥ 25% of families living below poverty level)

^d^Census tracts with < 50% of non-Hispanic White residents

## Discussion

Childhood exposure to lead continues to be a significant environmental public health concern. National data documents that lead exposure disproportionately burdens non-Hispanic Black children, children in families living below the federal poverty level, and children living in older housing [23]. Our study revealed that Milwaukee County neighborhoods do not have equal risk of childhood lead exposure. Consistent with previous studies [5–11,23–27], we document socioeconomic and racial inequities in childhood lead exposure at a neighborhood level. However, our results also reveal that risk of elevated blood lead level is greatest in neighborhoods with multiple risk factors, including high poverty, low home ownership, and majority non-White residents. Our study illustrated a neighborhood level socioeconomic inequity in mean childhood blood lead levels exacerbated by racial/ethnic composition, demonstrating that community lead prevention efforts should be informed by the intersection of multiple indicators of sociodemographic disadvantage, including home ownership, poverty, and race/ethnicity.

Current childhood lead prevention efforts in Milwaukee are limited by several factors. Wisconsin has a decentralized public health system with 92 local city and county health departments [43]. Milwaukee County alone has eleven local health departments, of which the City of Milwaukee’s Health Department is the largest [44]. Additionally, due to the high number of children residing in the City of Milwaukee with elevated blood lead levels and the limited local resources for lead prevention, case management and environmental assessments to mitigate lead exposure are only provided to children with one venous blood lead level test ≥ 20 μg/dL or two venous blood lead level tests ≥ 15 μg/dL drawn at least 90 days apart [22]. These factors contribute to the limited and fragmented lead prevention services available to Milwaukee’s children. Indeed, from 2014–2016, 10,004 children in the City of Milwaukee had elevated blood lead levels according the CDC but were below the City of Milwaukee’s level of action, and did not receive case management or mitigation services, despite knowing that no level of lead is safe. The inequity in risk of childhood lead exposure is compounded by lack of equal access to case management and mitigation services for affected children in Milwaukee, that but for their location of residence, they would have received. This underscores the importance of identifying “upstream” social determinants of lead exposure to be targeted by primary and primordial prevention efforts in Milwaukee and other cities struggling with the dual problem of high prevalence of lead exposed children and scarce resources.

Further, Milwaukee’s old housing stock and stark segregation poses a challenge for lead exposure prevention [14,16]. While the age of housing stock is an important risk factor for lead exposure, old housing is distributed throughout Milwaukee County, but socioeconomic disadvantage and race/ethnicity are not. Areas with high poverty and low home ownership represent neighborhoods where residents may lack the ability and agency to remediate sources of lead exposure in their homes. Renters living in poverty may face barriers in testing lead sources or receiving lead remediation due to financial costs, but also may be less likely to initiate testing or remediation with their landlord due to inherent power differentials, rental contract restrictions, or threat of eviction. In Wisconsin, landlords of rental properties built before 1978 are not required to test for lead-based paint and are only required to disclose known lead-based paint hazards to potential tenants [45]. Wisconsin property owners are only required to eliminate lead-based paint hazards if the Department of Health Services initiates a property investigation report to do so upon notice that the property is home to a blood lead poisoned child under the age of 6 [46]. Alternatively, homeowners not living in poverty may have the financial means and agency to test and remediate lead sources in their home. Milwaukee neighborhood racial/ethnic composition magnify socioeconomic disparities in childhood lead exposure. Our results revealed that 56% of majority non-White census tracts in Milwaukee County were classified as high socioeconomic disadvantage, whereas only 7% of majority White census tracts were classified as high socioeconomic disadvantage. Since economic security is inherently linked to the historical legacy of racial discrimination within the housing market, understanding how these power dynamics operate within the housing market to influence childhood lead exposure is a key area for future research.

There are some limitations to the generalizability of the study results. Census tract mean childhood blood lead levels are only representative of the children who were tested between 2014 and 2016. While the mean childhood blood lead testing penetrances are to be interpreted with caution due to the limitations in using the 2010 US Census Bureau child population estimates, the mean childhood blood lead testing penetrance estimated for the census tracts within the City of Milwaukee (69.32%) is comparable to the 12 to 35-month testing rate reported by the City of Milwaukee in 2016 (64.7%) [47]. The large sample size and relatively high screening rate increases confidence that the average childhood blood lead levels analyzed in this study are representative of Milwaukee children tested for blood lead levels.

Another limitation is the potential for measurement error from capillary blood samples, as 70% of individual childhood blood lead level data points analyzed were recorded as capillary blood samples. While capillary tests are sensitive to environmental contamination, there is no way of knowing the direction of bias from capillary samples. Capillary sampling has been shown to have slightly lower sensitivity to venous sampling, although its specificity is comparable to venous sampling and its convenience has enhanced screening efforts [48]. However, due to the limitations of capillary samples, we performed a sensitivity analysis to quantify the impact of potential environmental contamination by subtracting 1 μg/dL off of all individual blood lead levels obtained from capillary tests. The sensitivity analysis estimates were only slightly attenuated, suggesting that our study inferences remain unchanged even if capillary blood lead levels were affected by environmental contamination.

Due to the cross-sectional nature of the data, it is possible that children were exposed to lead in different geographic locations than the neighborhood reported at the time of the blood sample. However, blood lead levels are an accurate snapshot of current lead exposure, reducing the threat to temporality. Additionally, the ecological design of the study limits the ability to assess individual-level associations, however, lead is a neighborhood exposure because of its social and geographic concentration [49]. Thus, it is necessary to document neighborhood-level inequities in lead exposure to understand the spatial context of its social determinants. Finally, census tracts were utilized as a proxy for neighborhoods for this analysis, which may not accurately represent informal neighborhood boundaries. Future research should explore smaller geographic levels of analysis that may more closely align with informal neighborhood boundaries in Milwaukee County to further tease apart residential segregation and childhood lead exposure. Additional multi-level analysis could be conducted to explore the associations between individual and neighborhood socioeconomic and racial characteristics in relation to childhood lead exposure.

Despite these limitations, this study utilized large surveillance datasets to estimate the intersectional effect of social determinants of childhood lead exposure, documenting neighborhood factors that co-occur and interact to influence childhood lead exposure. Showing the synergistic effects of social determinants on childhood lead exposure at a neighborhood level strengthens the public health knowledge base on the social patterning of environmental exposures and informs primary prevention efforts to reduce childhood lead exposure. Secondary prevention of childhood lead exposure, the identification and management of childhood blood lead levels after a child has already been exposed to lead, is not sufficient to eliminate the disproportionate burden of childhood lead exposure in vulnerable populations [23]. Primary prevention of childhood lead exposure, targeting the conditions that cause lead exposure before a child is exposed, is the most effective approach to mitigating inequities in lead exposure [23]. While Milwaukee is one of many rustbelt metropolitan areas with lead concerns, it has yet to establish a formal infrastructure that builds the capacity for primary lead prevention through policy change and coalition, like the city of Rochester, New York has done [50]. To tackle the link between neighborhood racial/ethnic composition and childhood lead exposure that this study illustrates, lead prevention efforts must align with equity-focused housing and economic policies. Lead-hazard reduction initiatives in residential housing have mandated “lead safe” certifications for rental units, additional lead disclosures, and expanded tenant rights [51,52]. These serve as models for local communities to implement primary prevention policies to mitigate childhood lead exposure, particularly in neighborhoods that are disproportionately burdened by childhood lead exposure. However, action to strengthen tenant protections or efforts to increase home ownership must also address the legacy of policies and practices that have historically marginalized lower income communities of color.

## Conclusions

Poverty, home ownership, and race/ethnicity at the neighborhood level co-occur and interact to produce inequities in childhood lead exposure. The risk of elevated childhood blood lead levels is greatest in majority non-White Milwaukee County neighborhoods with high poverty and low home ownership. By illustrating the intersection of neighborhood-level social patterns associated with childhood lead exposure, this study provides support for lead prevention efforts to align with equity-focused housing and economic policies to target primary prevention in neighborhoods disproportionately burdened by childhood lead exposure.

## Supporting information

S1 Table Descriptive statistics of Milwaukee County census tracts by childhood blood lead levels (n = 215). Milwaukee County census tract-level distributions were averaged across all census tracts, displaying the average percent or mean value by childhood blood lead levels.
(PDF)

S2 Table Descriptive statistics of Milwaukee County census tracts by intersection of housing tenure and poverty level (n = 215). Milwaukee County census tract-level distributions were averaged across all census tracts, displaying the average percent or mean value by intersection of housing tenure and poverty level.
(PDF)

S3 Table Socioeconomic and racial/ethnic composition of Milwaukee County census tracts by average childhood blood lead level (n = 215). Socioeconomic and racial/ethnic characteristics of Milwaukee County census tracts with mean childhood blood lead level ≥ 5 μg/dL “Elevated” and < 5 μg/dL “Not Elevated”.
(PDF)

S1 File Linear regression analyses results (n = 168). Estimated regression coefficients, standard errors, p-values, confidence intervals, and R-square for linear regression models.
(PDF)
