# Supplementary material for: The intersectional effect of poverty, home ownership, and racial/ethnic composition on mean childhood blood lead levels in Milwaukee County neighborhoods
Source: PLoS One. 2020 Jun 19;15(6):e0234995. doi: 10.1371/journal.pone.0234995 (PMC7304591; doi:10.1371/journal.pone.0234995)
Supplement: S1 Table — Milwaukee County census tract-level distributions were averaged across all census tracts, displaying the average percent or mean value by childhood blood lead levels. (PDF) [file pone.0234995.s001.pdf]

	"Elevated" Census tracts with mean childhood blood lead level ≥ 5µg/dL	"Not Elevated" Census tracts with mean childhood blood lead level < 5µg/dL	
	N=60	N=155	
	Census Tract-Level Average[a]		p-value[b]
Wisconsin Surveillance Data			
Sex of Children Tested			
Female	49.15%	48.84%	0.7130
Male	50.82%	51.09%	0.7130
Unknown	0.03%	0.07%	
Age of Children Tested (in years)	2.58 (0.12)	2.19 (0.36)	<0.0001
Race/Ethnicity of Children Tested			
White	2.75%	24.62%	<0.0001
Black	69.52%	34.02%	<0.0001
Hispanic	17.30%	22.84%	0.0064
Other[c]	4.56%	6.73%	<0.0001
Unknown	5.88%	11.79%	<0.0001
Lead Test Sample Year			
2014	47.15%	48.33%	0.1391
2015	28.73%	29.94%	0.0868
2016	24.11%	21.73%	<0.0001
Lead Test Sample Type			
Capillary	66.35%	68.44%	0.0840
Venous	32.77%	30.65%	0.0808
Unknown	0.87%	0.92%	0.8116
2012 - 2016 American Community Survey 5-Year Estimates			
Race/Ethnicity			
Non-Hispanic White	10.77%	46.18%	<0.0001
Non-Hispanic Black or African American	67.16%	29.74%	<0.0001
Hispanic or Latino	15.34%	17.26%	0.0191
Other[d]	6.66%	6.79%	0.0191
Families Living Below the Federal Poverty Level	40.84%	19.37%	<0.0001
Housing Tenure			
Owner Occupied Housing	28.87%	45.97%	<0.0001
Renter Occupied Housing	71.13%	54.03%	<0.0001
Educational Attainment			
Less than HS Diploma[e]	26.73%	15.03%	<0.0001
HS Diploma/GED/equivalent[f]	33.48%	28.28%	0.0002
Beyond HS Diploma[g]	39.80%	56.69%	<0.0001
Housing Age			
Built Before 1950	73.50%	43.66%	<0.0001
Built 1950 or After	26.50%	56.34%	<0.0001

[a]Census Tract-Level Averages: Census tract-level distributions were averaged across all census tracts to generate the average percent or mean value

[b]P-value of two-tailed t-test or Wilcoxon two-sample tests, depending on the distribution

[c]Includes American Indian/Alaska Native, Asian, Hawaiian/pacific islander, Multiple races, and other

[d]Includes Non- Hispanic Asian, Non-Hispanic American Indian/Alaska Native, and Non-Hispanic, 2 or more races/ other race

[e]Includes estimates for population 25 years and older with no schooling, nursery school, kindergarten, and grade 1 -12 but no high school diploma

[f]Includes estimates for population 25 years and older with a high school diploma or GED or equivalent

[g]Includes estimates for population 25 years and older with some college/less than 1 year, some college/1 or more years no degree, Associate's degree, Bachelor's degree, Master's degree, Professional school degree, or Doctorate degree
