# Supplementary material for: The intersectional effect of poverty, home ownership, and racial/ethnic composition on mean childhood blood lead levels in Milwaukee County neighborhoods
Source: PLoS One. 2020 Jun 19;15(6):e0234995. doi: 10.1371/journal.pone.0234995 (PMC7304591; doi:10.1371/journal.pone.0234995)
Supplement: S2 Table — Milwaukee County census tract-level distributions were averaged across all census tracts, displaying the average percent or mean value by intersection of housing tenure and poverty level. (PDF) [file pone.0234995.s002.pdf]

	Census Tracts with High Home Ownership[a] & Low Poverty[c]	Census Tracts with Low Home Ownership[b] & Low Poverty[c]	Census Tracts with High Home Ownership[a] & High Poverty[d]	Census Tracts with Low Home Ownership[b] & High Poverty[d]	
	N=85	N=29	N=18	N=83	
	Census Tract-Level Average[e]				p-value[f]
Wisconsin Surveillance Data					
Sex of Children Tested					
Female	48.71%	49.43%	47.88%	49.20%	0.5995
Male	51.23%	50.43%	52.09%	50.77%	0.6382
Unknown	0.06%	0.13%	0.03%	0.03%	0.4229
Age of Children Tested (in years)	2.09 (0.32)	2.03 (0.34)	2.51 (0.11)	2.56 (0.20)	<0.0001
Race/Ethnicity of Children Tested					
White	31.61%	31.94%	3.54%	3.67%	<0.0001
Black	29.66%	26.46%	57.68%	61.65%	<0.0001
Hispanic	19.74%	17.58%	26.73%	23.01%	0.3059
Other[g]	6.41%	9.20%	5.46%	4.90%	0.1044
Unknown	12.58%	14.83%	6.59%	6.77%	<0.0001
Lead Test Sample Year					
2014	48.59%	47.74%	48.98%	47.27%	0.1373
2015	30.22%	31.13%	28.02%	28.78%	0.0172
2016	21.09%	21.13%	23.00%	23.94%	<0.0001
Lead Test Sample Type					
Capillary	70.02%	60.08%	70.04%	67.88%	0.4243
Venous	28.97%	39.52%	28.67%	31.23%	0.4091
Unknown	1.02%	0.40%	1.29%	0.89%	0.7637
2012 - 2016 American Community Survey 5-Year Estimates					
Race/Ethnicity					
Non-Hispanic White	54.30%	56.72%	16.45%	15.02%	<0.0001
Non-Hispanic Black or African American	25.34%	24.91%	52.65%	58.02%	<0.0001
Hispanic or Latino	13.26%	12.22%	24.57%	20.16%	0.0230
Other[h]	7.07%	6.09%	6.25%	6.77%	0.7787
Families Living Below the Federal Poverty Level	12.01%	12.47%	30.62%	42.40%	<0.0001
Housing Tenure					
Owner Occupied Housing	59.88%	28.45%	47.94%	25.06%	<0.0001
Renter Occupied Housing	40.12%	71.55%	52.06%	74.95%	<0.0001
Educational Attainment					
Less than HS Diploma[i]	11.64%	11.73%	22.16%	26.57%	<0.0001
HS Diploma/GED/equivalent[j]	28.33%	21.96%	35.48%	32.64%	0.0001
Beyond HS Diploma[k]	60.03%	66.30%	42.36%	40.80%	<0.0001
Housing Age					
Built Before 1950	41.19%	52.76%	56.52%	61.78%	<0.0001
Built 1950 or After	58.81%	47.24%	43.48%	38.22%	<0.0001

[a]Census tracts with ≥ 40% of occupied housing that is owned

[b]Census tracts with < 40% of occupied housing that is owned

[c]Census tracts with < 25% of families living below poverty level

[d]Census tracts with ≥ 25% of families living below poverty level

[e]Census Tract-Level Averages: Census tract-level distributions were averaged across all census tracts to generate the average percent or mean value

[f]P-value from f-test or chi-square test

[g]Includes American Indian/Alaska Native, Asian, Hawaiian/pacific islander, Multiple races, and other

[h]Includes Non- Hispanic Asian, Non-Hispanic American Indian/Alaska Native, and Non-Hispanic, 2 or more races/ other race

[i]Includes estimates for population 25 years and older with no schooling, nursery school, kindergarten, and grade 1 -12 but no high school diploma

[j]Includes estimates for population 25 years and older with a high school diploma or GED or equivalent

[k]Includes estimates for population 25 years and older with some college/less than 1 year, some college/1 or more years no degree, Associate's degree, Bachelor's degree, Master's degree, Professional school degree, or Doctorate degree
