# Supplementary material for: The intersectional effect of poverty, home ownership, and racial/ethnic composition on mean childhood blood lead levels in Milwaukee County neighborhoods
Source: PLoS One. 2020 Jun 19;15(6):e0234995. doi: 10.1371/journal.pone.0234995 (PMC7304591; doi:10.1371/journal.pone.0234995)
Supplement: S3 Table — Socioeconomic and racial/ethnic characteristics of Milwaukee County census tracts with mean childhood blood lead level ≥ 5 μg/dL “Elevated” and < 5 μg/dL “Not Elevated”. (PDF) [file pone.0234995.s003.pdf]

	**"Elevated"**<br>Census tracts with mean childhood blood lead levels ≥ 5 µg/dL	
	Majority White (N=2)	Majority Non-White (N=58)
	N (%)	
High Home Ownership[a] & Low Poverty[c]	1 (50.00%)	3 (5.17%)
Low Home Ownership[b] & Low Poverty[c]	0 (0.00%)	3 (5.17%)
High Home Ownership[a] & High Poverty[d]	0 (0.00%)	5 (8.62%)
Low Home Ownership[b] & High Poverty[d]	1 (50.00%)	47 (81.03%)

	**"Not Elevated"**<br>Census tracts with mean childhood blood lead levels < 5 µg/dL	
	Majority White (N=74)	Majority Non-White (N=81)
	N (%)	
High Home Ownership[a] & Low Poverty[c]	52 (70.27%)	29 (35.80%)
Low Home Ownership[b] & Low Poverty[c]	18 (24.32%)	8 (9.88%)
High Home Ownership[a] & High Poverty[d]	0 (0.00%)	13 (16.05%)
Low Home Ownership[b] & High Poverty[d]	4 (5.41%)	31 (38.27%)

[a]Census tracts with ≥ 40% of occupied housing that is owned

[b]Census tracts with < 40% of occupied housing that is owned

[c]Census tracts with < 25% of families living below poverty level

[d]Census tracts with ≥ 25% of families living below poverty level
