# Supplementary material for: The intersectional effect of poverty, home ownership, and racial/ethnic composition on mean childhood blood lead levels in Milwaukee County neighborhoods
Source: PLoS One. 2020 Jun 19;15(6):e0234995. doi: 10.1371/journal.pone.0234995 (PMC7304591; doi:10.1371/journal.pone.0234995)
Supplement: S1 File — Estimated regression coefficients, standard errors, p-values, confidence intervals, and R-square for linear regression models. (PDF) [file pone.0234995.s004.pdf]

## Unadjusted Model 1

| Parameter | Estimate | Standard Error | P-value | 95% Confidence Limits | | R-sqaure= 0.477273 |
|---|---|---|---|---|---|---|
| Majority White, Low Socioeconomic Disadvantage | Ref. | | | | | |
| Majority White, High Socioeconomic Disadvantage | 0.527218626 | 0.45464478 | 0.2479 | -0.370493204 | 1.424930456 | |
| Majority Non-White, Low Socioeconomic Disadvantage | 0.893573553 | 0.21755932 | <.0001 | 0.463995162 | 1.323151945 | |
| Majority Non-White, High Socioeconomic Disadvantage | 2.078742837 | 0.17299421 | <.0001 | 1.737159796 | 2.420325878 | |
| Intercept | 3.249716045 | 0.1334883 | <.0001 | 2.986138776 | 3.513293314 | |

## Adjusted Model 2

| Parameter | Estimate | Standard Error | P-value | 95% Confidence Limits | | R-square= 0.605310 |
|---|---|---|---|---|---|---|
| Majority White, Low Socioeconomic Disadvantage | Ref. | | | | | |
| Majority White, High Socioeconomic Disadvantage | 0.484464432 | 0.39631331 | 0.2233 | -0.298105585 | 1.26703445 | |
| Majority Non-White, Low Socioeconomic Disadvantage | 0.74782062 | 0.19068174 | 0.0001 | 0.371295771 | 1.124345469 | |
| Majority Non-White, High Socioeconomic Disadvantage | 1.668890571 | 0.16097225 | <.0001 | 1.351030797 | 1.986750346 | |
| Majority Old Housing | 1.029987397 | 0.14164399 | <.0001 | 0.750293679 | 1.309681115 | |
| Intercept | 2.880475281 | 0.12694664 | <.0001 | 2.62980332 | 3.131147241 | |

## Final Adjusted Model 3

| Parameter | Estimate | Standard Error | P-value | 95% Confidence Limits | | R-square= 0.613320 |
|---|---|---|---|---|---|---|
| Majority White, Low Socioeconomic Disadvantage | Ref. | | | | | |
| Majority White, High Socioeconomic Disadvantage | 0.369161878 | 0.3984827 | 0.3556 | -0.417728205 | 1.156051961 | |
| Majority Non-White, Low Socioeconomic Disadvantage | 0.850260768 | 0.19740501 | <.0001 | 0.460441972 | 1.240079565 | |
| Majority Non-White, High Socioeconomic Disadvantage | 1.777212899 | 0.17040998 | <.0001 | 1.440701616 | 2.113724181 | |
| Majority Old Housing | 0.971790444 | 0.1441752 | <.0001 | 0.687085403 | 1.256495485 | |
| Number of Children | -0.000768585 | 0.00041957 | 0.0688 | -0.001597108 | 0.000059938 | |
| Intercept | 3.105753047 | 0.17609478 | <.0001 | 2.758015913 | 3.45349018 | |
